# Spontaneous formation of fluid escape pipes from subsurface reservoirs

**DOI:** 10.1038/s41598-018-29485-5

**Published:** 2018-07-24

**Authors:** Ludovic Räss, Nina S. C. Simon, Yury Y. Podladchikov

**Affiliations:** 1 Institute of Earth Sciences, University of Lausanne, Géopolis, 1015 Lausanne Switzerland; 2 Swiss Geocomputing Centre, University of Lausanne, Géopolis, 1015 Lausanne Switzerland; 3 SignificaNS, Oslo, Norway

## Abstract

Ubiquitous observations of channelised fluid flow in the form of pipes or chimney-like features in sedimentary sequences provide strong evidence for significant transient permeability-generation in the subsurface. Understanding the mechanisms and dynamics for spontaneous flow localisation into fluid conductive chimneys is vital for natural fluid migration and anthropogenic fluid and gas operations, and in waste sequestration. Yet no model exists that can predict how, when, or where these conduits form. Here we propose a physical mechanism and show that pipes and chimneys can form spontaneously through hydro-mechanical coupling between fluid flow and solid deformation. By resolving both fluid flow and shear deformation of the matrix in three dimensions, we predict fluid flux and matrix stress distribution over time. The pipes constitute efficient fluid pathways with permeability enhancement exceeding three orders of magnitude. We find that in essentially impermeable shale (10^−19^ m^2^), vertical fluid migration rates in the high-permeability pipes or chimneys approach rates expected in permeable sandstones (10^−15^ m^2^). This previously unidentified fluid focusing mechanism bridges the gap between observations and established conceptual models for overcoming and destroying assumed impermeable barriers. This mechanism therefore has a profound impact on assessing the evolution of leakage pathways in natural gas emissions, for reliable risk assessment for long-term subsurface waste storage, or CO_2_ sequestration.

## Introduction

**Figure 1:**
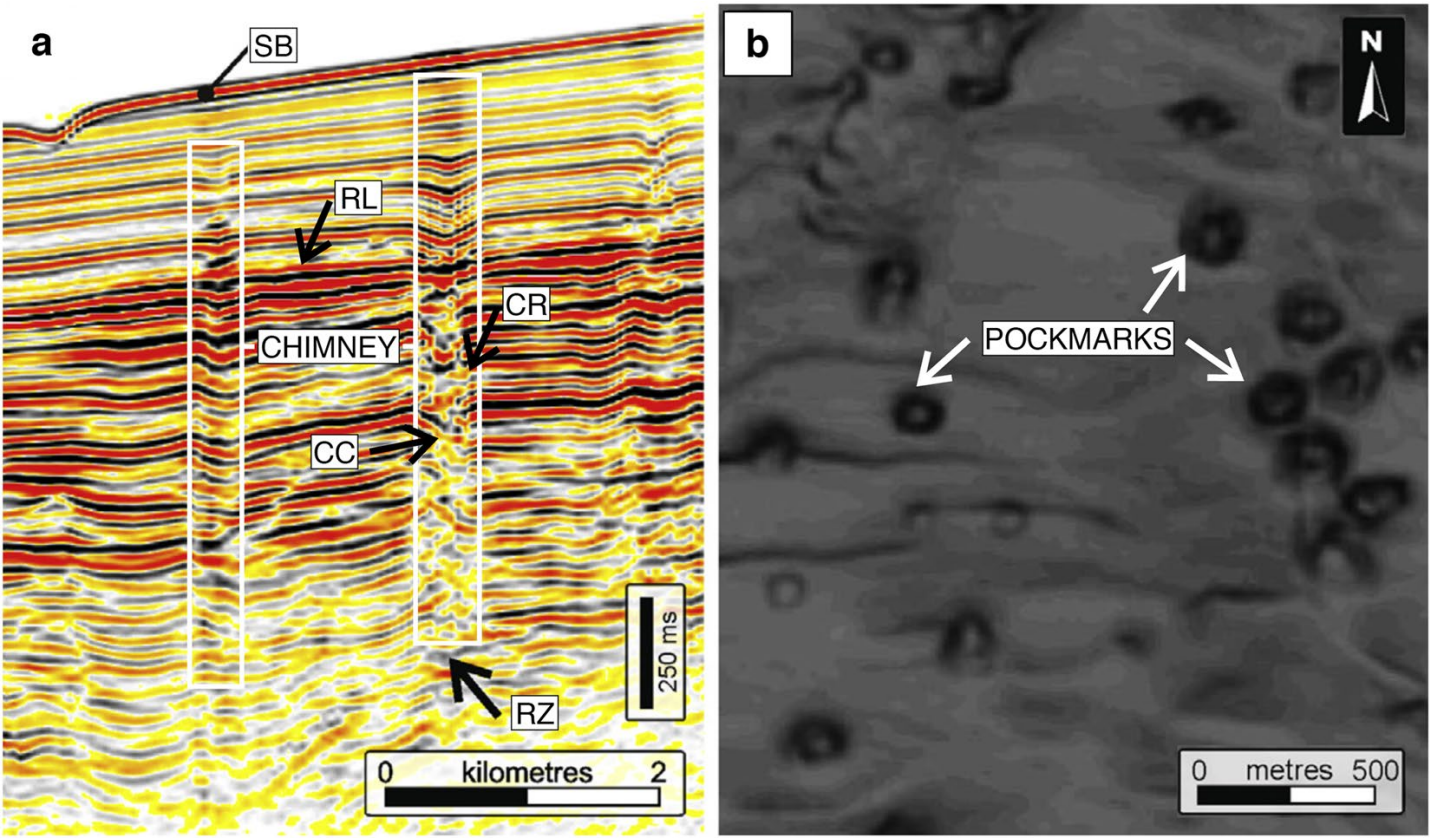
Seismic expression of chimneys and pockmarks. Figure modified from Cartwright and colleagues^11,22^. (**a**) Vertical seismic profile through fluid migration pathways from offshore Namibia. SB = seabed, RL = reflective horizontal sedimentary layer, CR = chimney downward-bending compacted rim, CC = chimney core, RZ = root zone and diffuse base of the chimney. (**b**) Horizontal slice through a group of chimneys displaying the typical circular craters or pockmarks.

**Figure 2:**
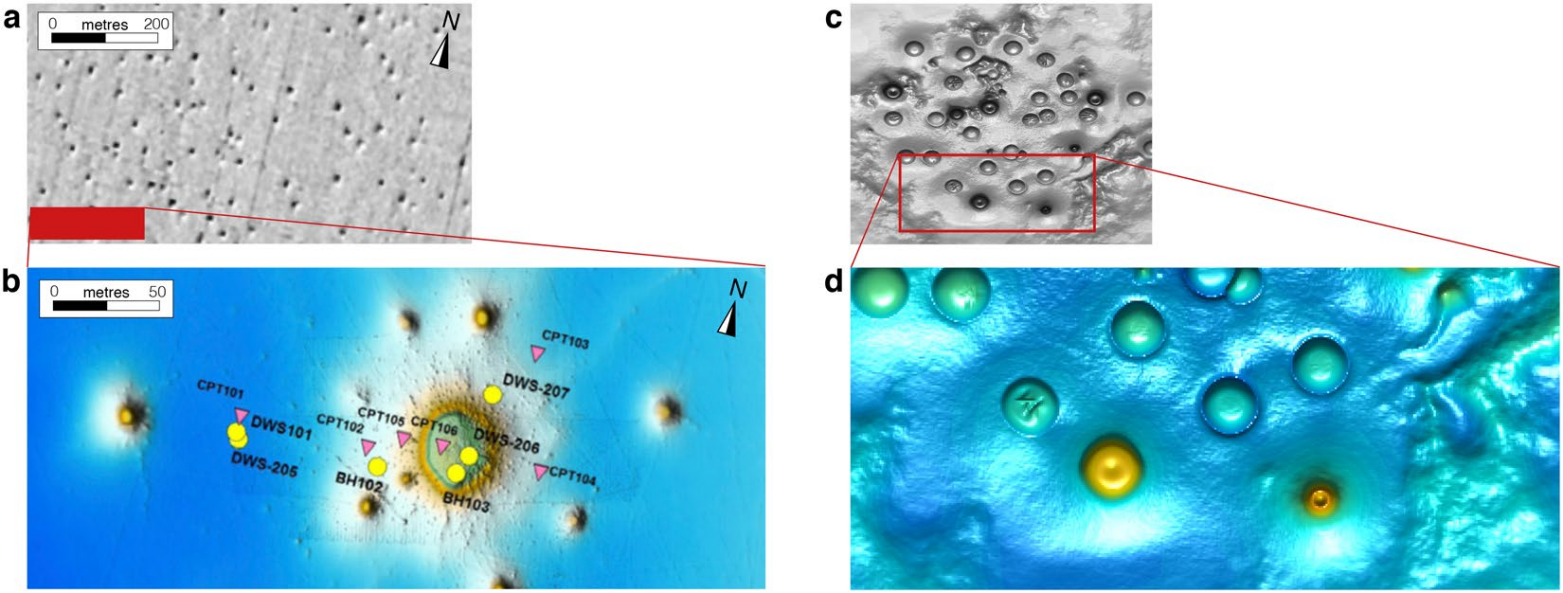
Comparison of the numerical results to pockmarks observed on the seafloor. (**a**) Natural data showing pockmarks on the seafloor in part of the Troll field area, offshore Norway. Figure modified from Mazzini and colleagues^15^. (**b**) Multibeam line across the red rectangular region from (**a**), displaying high-resolution seafloor mapping of pockmarks. (**c**) Numerical result from the simulation described in the main text (Fig. 5b,d), reoriented to fit the natural data aspect ratios. (**d**) Magnification of a specific region of the numerical model results (red rectangle) showing crater distribution, size variation and topography.

Fluid and melt expulsion from porous rocks occurs at many scales on Earth, from the mantle to the shallow subsurface^1–10^. In the shallow subsurface, distinct features are observed^11–16^ of vertical chimneys associated with localised continuous or episodic buoyancy-driven fluid flow. These vertical fluid migration pathways are located in most of the studied sedimentary basins. As these regions are of economic interest, they are widely screened with geophysical methods, which provide excellent data that clearly exhibit fluid migration pathways in three dimensions^17^. These features are particularly recognisable by their specific signature on seismic cross-sections (Fig. 1a), while pockmarks and circular-shaped craters (Fig. 1b) are their related expression on the seafloor (Fig. 2a,b). These focussed flow pathways provide an important and efficient transport mechanism for fluid migration, yet the physical controls on their formation are not well understood and still debated^18–20^. We propose hydro-mechanical coupling as the dominant underlying physical process for the formation of high-permeability pathways, based on the following observations: (1) Vertical chimneys occur in various sedimentary basin lithologies^12,14,21^ and appear unaffected by rock composition, refuting chemical reactions as the main formation mechanism. (2) The vertical chimneys develop through existing formations and are not influenced by sedimentary layering or structural features such as faults^13,21,22^. These observations also suggest that they mainly do not form by reactivation of pre-existing structures, but are the outcome of a self-sustaining dynamic process independent of the inherited geological setting. However, pipes or chimneys may depart from fault planes or root at a similar stratigraphic level. In addition, we do not exclude chemical reactions and hydro-chemo-mechanical coupling to contribute to the generation and propagation of pore fluids^23–25^. Since chimney formation cannot be inferred from static models nor geological history, there is a need to develop predictive models to resolve their spontaneous formation and to better constrain the parameters that govern their propagation.

**Figure 3:**
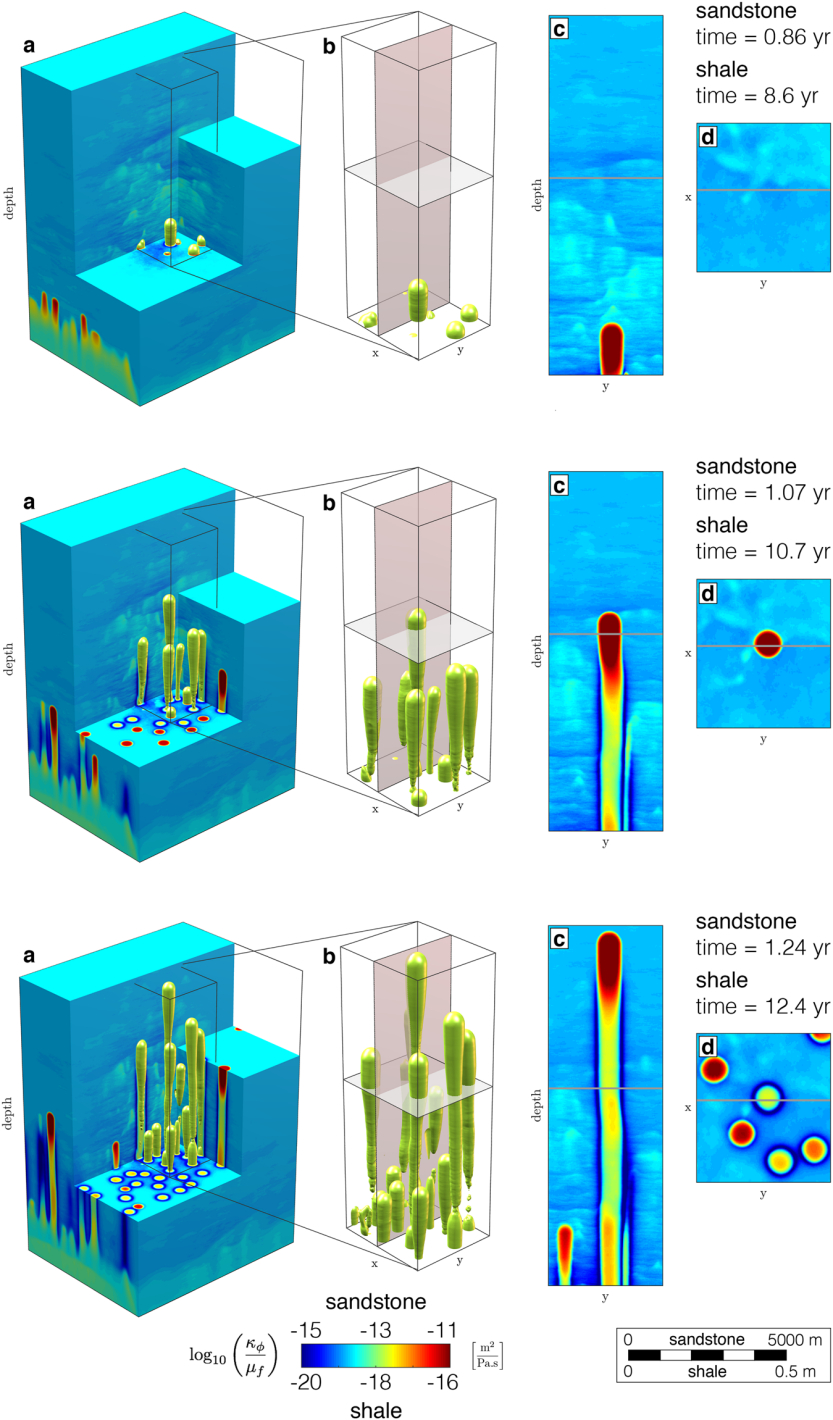
High-permeability chimney genesis out of a source region (reservoir) in three dimensions. Colour plot (logarithmic scale) of dynamic permeability $({\kappa }_{\varphi }/{\mu }_{f})$ for two different lithologies, conductive sandstone and impermeable shale. Contoured values show a 1.5 order of magnitude increase in $({\kappa }_{\varphi }/{\mu }_{f})$ representative for the chimneys. (**a**) Insight into the hydro-mechanical model unveiling the existence of high-permeability chimneys as tubular shaped features in three dimensions. (**b**) Enlargement of the centre of the model, selectively displaying the contoured chimneys. (**c**) Vertical two-dimensional slice of (**b**) displaying a colour plot of the permeability field of an isolated chimney. (**d**) Horizontal slice of (**b**) displaying a colour plot of the permeability field, resulting in rounded craters or pockmarks. Effective permeability, time and length scale are given for both permeable sandstones and low-permeability shales^37^.

We address this issue by considering a poro-visco-elastic model of rock deformation and flow to quantify the process of channelised fluid migration associated with the deformation of permeable rocks^26^. We use a continuum mechanics model to compute the porous matrix deformation using a non-linear bulk and shear rheology. Resolving the mechanics is required to calculate the total pressure, strain rates and corresponding stresses, which implies evaluating the entire deviatoric stress tensor. We couple the mechanical solver with a Darcy flow solver and calculate the fluid fluxes using the non-linear Carman-Kozeny relation^27^ for dynamic permeability. We streamline a supercomputing approach to resolve the coupled fluid-rock interactions in high resolution^28^. This hydro-mechanical model permits us to predict high-permeability chimney formation and propagation, and the stress distribution in the deforming poro-visco-elastic matrix in three dimensions (Fig. 3). The fully resolved hydro-mechanical coupling generates and propagates solitary waves^29–32^ of porosity within a specific parameter range. A pressure sensitive viscous bulk rheology further triggers a significant flow focussing mechanism, ‘decompaction weakening’^1^, at the top of the solitons. The usual spherically shaped solitons^32,33^ turn into elongated chimney-shaped features^28^ (Fig. 3). Solitary waves have been proposed as mechanism for enhanced fluid transport in deep crustal and mantle rocks, as well as for primary hydrocarbon and methane migration from source rocks into sedimentary basins^1,4,25,31,32,34,35^. These deep rock formations are generally considered to exhibit viscous or creep behaviour, in contrast to shallow sedimentary rocks, which are believed to deform in a more elastic and brittle way. However, recent laboratory experiments on major sedimentary reservoir rock types indicate that time-dependent deformation must be considered^36,37^. The clay content of sediments strongly enhances creep and self-sealing capabilities while hindering the propagation of brittle fractures^37^. Thus, ‘soft’ clay-rich rocks are regarded as natural barriers^21,37,38^ and are recommended as caprock for storage operations. However, although brittle processes are inhibited, a time-dependent creep rheology may lead to localised flow of the sedimentary material in response to applied stress, proportional to bulk viscosity values in the range of 10^16^–10^13^ Pa.s^37,39^.

## Results

**Figure 4:**
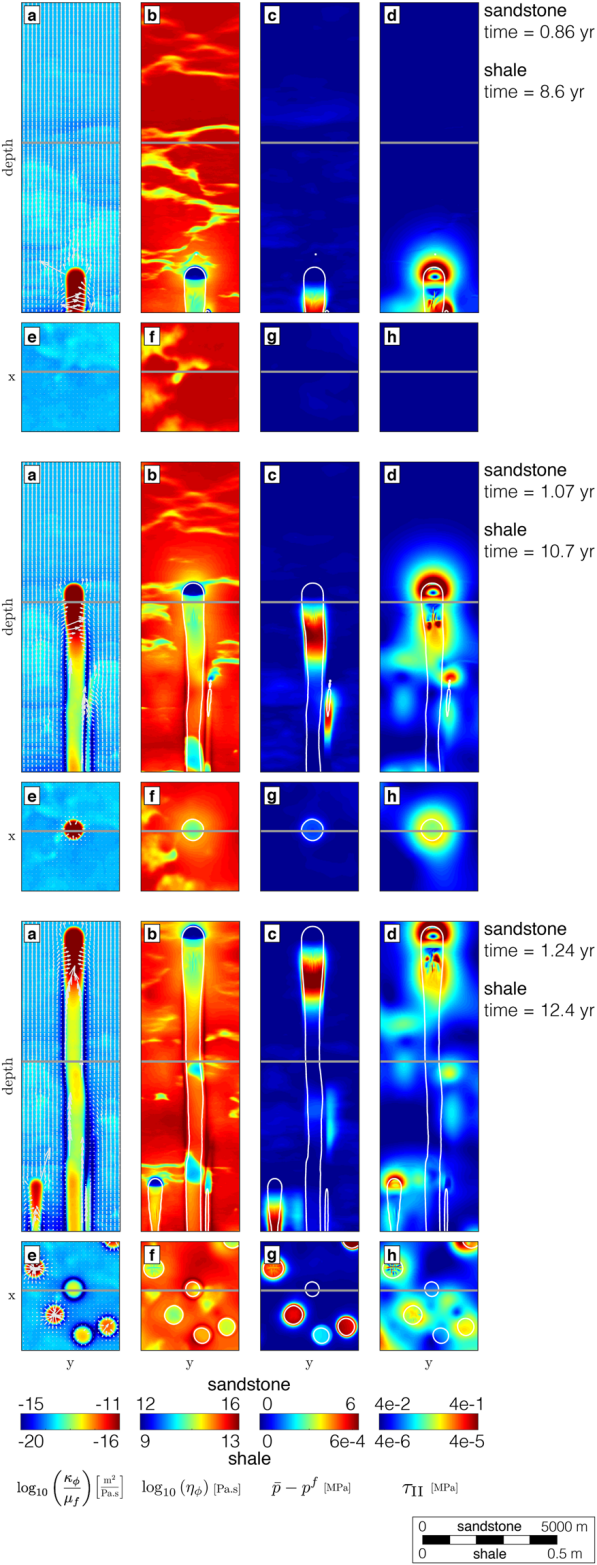
Chimney formation mechanism. Three successive time laps of two-dimensional vertical (**a**–**d**) and horizontal (**e**,**f**) slices from Fig. 1b. (**a**,**e**) Dynamic permeability (logarithmic scale) field. The white arrows represent the fluid flux vectors, scaled by the maximal flux over time and directed into the chimney in the local drainage area, showing flux from outside to inside the chimneys. (**b**,**f**) Strain rate-dependent non-linear bulk viscosity values (logarithmic scale). (**c**,**g**) Effective pressure ($\bar{p}-{p}^{f}$) distribution. (**d**,**h**) Shear stress deformation magnitude (second invariant of the deviatoric stress tensor). Results are scaled for low-permeable shale (${\kappa }_{{\rm{shale}}}={10}^{-19}$ m^2^) and permeable sandstone (${\kappa }_{{\rm{sand}}}={10}^{-14}\,$m^2^) and display downward-bending compacted chimney rims, permeable chimney cores and circular pockmarks; the characteristic chimney attributes observed in nature. White contour lines (**b**–**d**,**f**–**h**) represent the chimney extend, characterised by a significant increase (1.5 order of magnitude) in dynamic permeability.

We show the spontaneous development of high-permeability (>3 orders of magnitude over background values) chimneys from a fluid-enriched source region in three dimensions (Fig. 3). The buoyant pore-fluid triggers local decompaction of the porous medium and enables upward-migration within self-organised chimneys. This contrasts with Darcian flow models in non-deforming porous media that predict diffusive fluid flow and spreading of fluids. The focussed flow patterns have a tubular shape (Fig. 3a,b), which can only be resolved by 3-D models. Both the vertical and horizontal 2-D expression (Fig. 3c,d) of the contoured highly permeable regions (Fig. 3b) reproduce natural seismic pipes or chimneys (Fig. 1) and pockmark observations (Fig. 2). The physical mechanism leading to chimney formation is a natural outcome of time-dependent creep deformation of the fluid-rich porous matrix interacting with the non-linear flow of the pore-fluid. The difference between total pressure and fluid pressure (Fig. 4c) affects the bulk viscosity distribution (Fig. 4b) in a non-linear way and leads to a significant permeability increase (Fig. 4a). The upward-migration of the highly permeable chimneys is sustained by active fluid drainage from the immediate surrounding regions, leading to localised compaction. The fluid flux vectors (white arrows) pointing inward into the pipes support this hypothesis (Fig. 4a,e). The resulting consolidated chimney rim is characterised by decreased permeability and increased viscosity values (Fig. 4a,b,e,f). This phenomenon may indicate that the consolidated rims correspond to the downward-bending horizontal reflectors diagnostic for chimney occurrence in seismic cross-sections (Fig. 1). The consolidated rim confines pressure deviations (Fig. 4c) and high fluid fluxes to within the chimney. Thus, pressure measurements outside the chimneys may show no significant perturbations. In contrast to pressure, the localised shear deformation may be detected outside of the chimneys, and the stress envelope runs slightly ahead of the propagating chimney. The second invariant of the deviatoric stress tensor (${\tau }_{II}$) quantifies the magnitude of shear deformation recorded by the porous matrix (Fig. 4d). Variations in stresses may be measurable with seismic methods and their increase in time may further trigger micro-seismic events. The transient fluid expulsion pulse induces irreversible alteration of the permeability and bulk viscosity distributions. These alterations may be responsible for the preservation of the dormant features recognisable in the field (Fig. 2). In contrast, variation in pressure and stresses are restricted to the transient deformation accompanying the fluid pulse.

**Figure 5:**
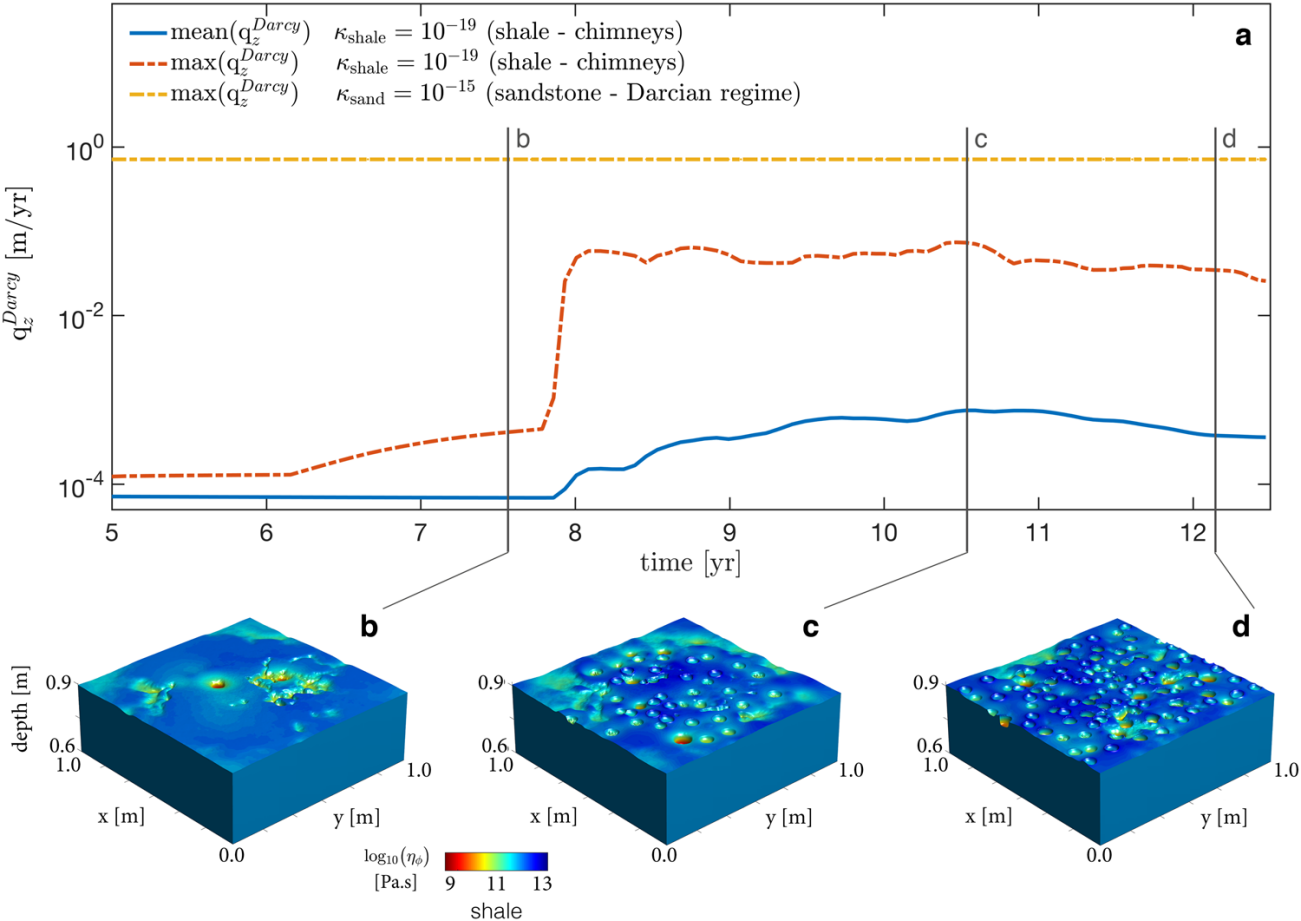
Fluid flux through a horizontal slice of 1 m^2^ of low-permeable shale (${\kappa }_{{\rm{shale}}}={10}^{-19}$ m^2^) located at 1 m above the source region showing corresponding typical circular craters or pockmarks. (**a**) Mean fluid flux $({\rm{mean}}({q}_{z}^{Darcy}))$ and maximal fluid flux $({\rm{\max }}({q}_{z}^{Darcy}))$ values in m/yr through the contributing area (1 m^2^) as a function of time for chimney populated shale. Comparison with fluid flux through 1 m^2^ of four orders of magnitude more permeable sandstone (${\kappa }_{{\rm{sand}}}={10}^{-15}$ m^2^) in pure diffusive Darcian regime without chimneys. Mean and maximal fluid flux values are identical for homogenous permeability distribution in sandstone. (**b**,**d**) Expression of craters resulting from flow focusing in high-permeability chimneys. Surface and colour plot of bulk viscosity (${\eta }_{\varphi }$) reflecting the geological records of contrasting material parameters, by analogy to Fig. 1b. (**b**) First chimney break through after 8 years. (**c**) Vertical flow peak after 10.5 years. (**d**) Lowered flux and dormant chimneys after 12 years.

Recording the fluid flux over time at a specific horizontal cross-section of the model confirms the ability of high-permeability chimneys to enhance vertical flow rates (Fig. 5). Using an initial permeability value of 10^−19^ m^2^, representative of typical clay-rich sealing sequences^37^, we predict that the formation of high-permeability chimneys leads to maximal flow rates up to 0.1 m/yr (mean flow rates of about 0.01 m/yr) through a 1 m^2^ horizontal cross-section of clay-rich shale (Fig. 5a). Thus, the preferential flow paths (chimneys) within clay-rich rocks enable flow rates of only one order of magnitude below expected diffusive Darcian flow through typical reservoir-quality sandstones with a permeability of 10^−15^ m^2^ (Fig. 5a). Prior to first chimney arrival, vertical fluid flux values are constant over time and reflect the four orders of magnitude discrepancy in background permeability values. At first chimney breakthrough (Fig. 5b), vertical fluid fluxes in the shale significantly increase and reach their maximal value over an extremely short time (Fig. 5c). Although the flow rates through the chimneys in the shale horizon decreased during the last year of the simulation (Fig. 5d), the highly conductive chimneys continuously enable high fluid fluxes at rates three orders of magnitude higher than the background.

## Discussion

Our results show that non-linear hydro-mechanical coupling provides a self-consistent mechanism for rapid and localised fluid expulsion even through a priori impermeable rocks. The chimneys are tubular features generated by transient fluid pulses that exhibit an increased permeability core and a compacted rim, which are preserved in the geological record. They display a characteristic size, spacing and propagation velocity. This stands in contrast to brittle fractures, which are planar features that occur instantaneously. Further, hydro-fractures require high fluid pressure to initiate and remain open, maintaining fluid conductivity. Hydro-fracturing results in a fluid pressure gradient that pushes fluid from the fracture interior into the host rock. Chimneys do not require an over-pressured source region and grow by collecting surrounding fluids into their under-pressured core. This self-sustained fluid collection process results in efficient fluid drainage (the white arrows in Fig. 4a,e), and the associated shear deformation generates a characteristic transient stress pattern.

Our results reproduce the natural observations of fluid migration conduits in many different locations (Figs 1 and 2) and geological settings^12,15,20,22,40^ and may have significant implications in other fields of geosciences such as hydrothermal systems^41,42^ or gas emissions in different tectonic environments^43^. Our findings suggest that the dominant mechanisms responsible for the spontaneous formation of fluid escape pipes in the subsurface are viscous creep of the porous matrix, decompaction weakening, and hydro-mechanical coupling. Numerical simulations predict that the activated creep leads to the rapid formation of chimneys in low-viscosity shales, expected to be natural flow barriers^13,21^. Thus, resolving the variations in flow patterns, viscous compaction and associated stresses is vital to evaluate storage potential and geological barrier integrity in oil and gas operations^13,44^ and waste sequestration^38^. Our results also suggest chimneys and pockmarks to be the expression of rapid migration of natural gas such as methane out of hydrate-rich sediments^10,15,45^. Understanding the migration mechanism is a prerequisite for accurately constraining these natural greenhouse gas fluxes towards the atmosphere, with potential implications for long-term climate modification, the evolution of Earth, and for society.

## Methods

### Summary

We utilised a thermodynamically consistent set of two-phase equations coupling non-linear Darcian flow with a mechanical poro-visco-elastic solver^26^. We adopted a Carman-Kozeny relation^27^ to capture the non-linear porosity-dependent permeability using a power-law exponent of 3. We utilised a viscous bulk and shear rheology for the matrix. The bulk viscosity ${\eta }_{\varphi }$ is inversely proportional to the porosity and reduced by a factor $R$ in the regions of fluid overpressure to account for decompaction weakening^1^:

1 $${\eta }_{\varphi }=\{\begin{array}{c}{\eta }_{C},{\rm{if}}\,{\rm{fluid}}\,{\rm{pressure}}\, < \,{\rm{total}}\,{\rm{pressure}}.\\ \frac{{\eta }_{C}}{R},{\rm{if}}\,{\rm{fluid}}\,{\rm{pressure}}\, > \,\text{total}\,\text{pressure}.\end{array}$$

The solid shear viscosity ${\mu }_{s}$ is a non-linear function of the strain rate and tends to a reference value for negligible strain rates.

We discretised the system of coupled hydro-mechanical partial differential equations using the Finite Difference Method on a regular Cartesian grid in 3-D. We reached an implicit solution of the stencil-based algorithm using an iterative approach in parallel on graphical processing units (GPUs). We utilised a high-resolution numerical grid of 500 × 500 × 1,000 grid points in x, y and z direction, respectively, required to accurately resolve chimney formation and propagation.

### Hydro-mechanical coupled model formulation

We employed a system of two-phase equations^26^ to model the formation and the evolution of high-porosity chimneys over time as a natural outcome of the coupling of fluid flow to the deformation of a viscous porous matrix. The mass balances for fluid and solid phases, assuming constant fluid and solid densities, were:

2 $${\nabla }_{k}{v}_{k}^{s}=-\frac{d\,\mathrm{log}(1-\varphi )}{dt},$$

3 $${\nabla }_{k}\varphi [{v}_{i}^{f}-{v}_{i}^{s}]=\frac{d\,\mathrm{log}(1-\varphi )}{dt},$$

where $\frac{d}{dt}=\frac{\partial }{\partial {\rm{t}}}+{v}_{k}^{s}{\nabla }_{i}$ is the material derivative with respect to the solid. The momentum balance equations for the matrix and the pore-fluid were:

4 $${\nabla }_{j}({\bar{\tau }}_{ij}-\bar{p}{\delta }_{ij})-\bar{\rho }{g}_{i}=0,$$

5 $$\varphi ({v}_{i}^{f}-{v}_{i}^{s})+\frac{{\kappa }_{\varphi }}{{\mu }_{f}}({\nabla }_{i}{p}_{i}^{f}+{\rho }^{f}{g}_{i})=0,$$

where ${\bar{\tau }}_{ij}$ are the components of the stress deviator, ${\delta }_{ij}$ is the Kronecker delta, ${g}_{i}$ are the components of the downward pointing gravity acceleration vector, $\varphi ({v}_{i}^{f}-{v}_{i}^{s})$ is the Darcy flux vector (the relative flux of the fluid relative to the solid), ${\mu }_{f}$ is the pore-fluid viscosity and $\bar{p}$, ${p}^{f}$ are the total and fluid pressures, respectively. The total porosity averaged density:

6 $$\bar{\rho }=(1-\varphi ){\rho }^{s}+\varphi {\rho }^{f},$$

includes constant solid and fluid densities ${\rho }^{s}$ and ${\rho }^{f}$, respectively. The Carman-Kozeny relation^27^ defines the porosity $\varphi$ dependent permeability ${\kappa }_{\varphi }$:

7 $${\kappa }_{\varphi }={\kappa }_{0}{(\frac{\varphi }{{\varphi }_{0}})}^{3},$$

where ${\kappa }_{0}$ is the reference permeability and ${\varphi }_{0}$ the reference porosity. The strain rate tensor and non-linear viscous creep rheology are expressed as:

8 $${\dot{{\epsilon }}}_{ij}=\frac{1}{2}({\nabla }_{i}{v}_{j}^{s}+{\nabla }_{j}{v}_{i}^{s})-\frac{1}{3}{\delta }_{ij}{\nabla }_{k}{v}_{k}^{s}=\frac{1}{2}A{{\tau }_{II}}^{n-1}{\bar{\tau }}_{ij},$$

where ${\dot{{\epsilon }}}_{ij}$ is the strain rate tensor, ${\bar{\tau }}_{ij}$ and ${\tau }_{II}$ are the deviatoric stress tensor and the square root of its second invariant, respectively, $n$ is the stress exponent and $A$ is a pre-exponential constant that is equal to the inverse of the solid shear viscosity in the linear viscous case $n=1$. The system is closed by a final constitutive equation accounting for viscous (de)compaction:

9 $${\nabla }_{k}{v}_{k}^{s}=-\frac{{p}_{e}}{{\eta }_{\varphi }(1-\varphi )},$$

where ${\eta }_{\varphi }$ is the bulk viscosity. The reference bulk compaction viscosity, ${\eta }_{C}$, at reference porosity ${\varphi }_{0}$ and $\bar{p}\gg {p}^{f}$ is:

10 $${\eta }_{C}=\frac{{\mu }_{s}}{C{\varphi }_{0}},$$

where $C$ is the pore geometry dependent coefficient. At other porosity and fluid pressure values, the bulk viscosity ${\eta }_{\varphi }$ is inversely proportional to the porosity and drops with the increase of the effective pressure ${p}_{e}=\bar{p}-{p}^{f}$ to account for decompaction weakening, parametrised by a hyperbolic tangent function in the numerical implementation:

11 $${\eta }_{\varphi }={\eta }_{C}\frac{{\varphi }_{0}}{\varphi }[1+\frac{1}{2}(\frac{1}{R}-1)(1+tanh[-\frac{{p}_{e}}{{\lambda }_{p}}])],$$

where ${\lambda }_{p}$ is the sharpness of the transition zone between the decompacting and the compacting regime and $R$ is a rheological constant^1^ that quantifies the ratio of compaction $({\eta }_{C})$ over decompaction bulk viscosity. The effective solid shear viscosity ${\mu }_{s}$ is a non-linear function of the strain rate and is implemented as:

12 $${\mu }_{s}=\frac{1}{2{A}^{\frac{1}{n}}{{\dot{{\epsilon }}}_{II}}^{\frac{n-1}{n}}+\frac{1}{{\mu }_{0}}},$$

where ${\dot{{\epsilon }}}_{II}$ is the square root of the second invariant of the deviatoric strain rate, $n$ is the power-law exponent (here $n=3$) and ${\mu }_{0}$ is the reference viscosity for negligible strain rates.

### Simulation details and initial conditions

**Figure 6:**
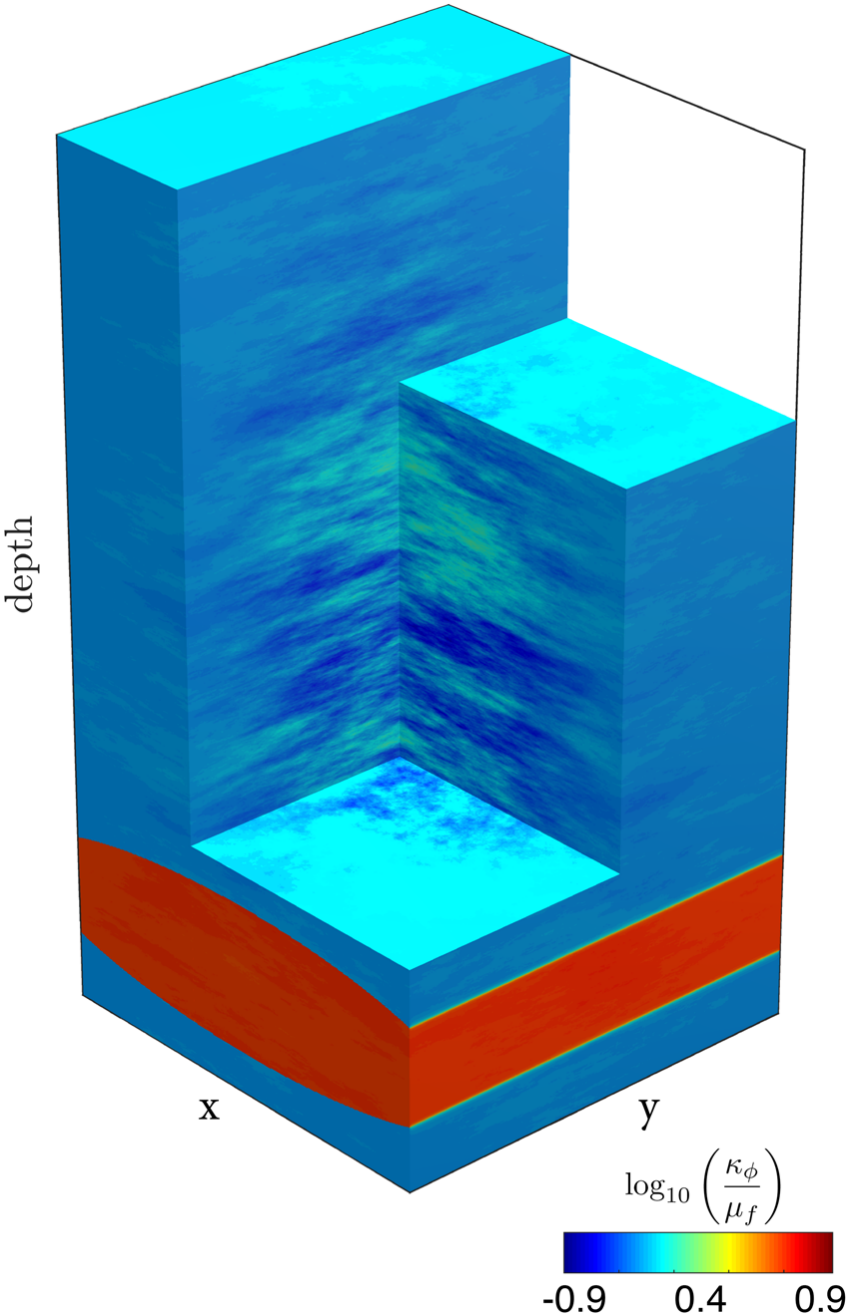
Initial conditions for the numerical simulation. Permeability distribution is set as an anisotropic Gaussian random field throughout the model. A cylindrical ellipse of close to one order of magnitude higher dynamic permeability values (logarithmic scale) compared to the background dimensionless value of 1 is located at ¼ from the bottom of the domain. Gravity is acting downwards and the pore fluid is twice as buoyant as the solid matrix.

The hydro-mechanical simulation initial configuration consists of a rectangular box with a dimensionless extent of 30 × 30 × 60 in the x, y (horizontal) and z (depth) directions, respectively. The initial porosity follows an anisotropic Gaussian random-field distribution, with a standard deviation equal to 1, and correlation lengths of 5, 5, 1 in the x, y and z directions, respectively. Further, a high-porosity cylindrical ellipse is located at the first ¼ of the domain height and represents a fluid-rich source region or reservoir. Permeability values within the source region were nine times higher compared to normalised background values of 1 (Fig. 6). The computational domain was subjected to the downward-pointing gravity field and affected by a background horizontal strike-slip shear-deformation of similar magnitude than buoyancy forces. The pore-fluid is twice as buoyant as the solid. The mechanical problem was solved using free-slip (no shear stress) boundary conditions on all sides of the box. For the fluid flow problem, we applied no flux boundary conditions on all vertical sides of the box, and fixed flux value at the bottom and top boundaries to satisfy the condition ${p}_{e}=0$ (no compaction or decompaction). A total number of 10,000 time steps was necessary to obtain the results. The dimensionless parameters used in the code (Table 1) allowed us to optimally converge the numerical simulation.

**Table 1:** Dimensionless physical values used in the computation.

Description	Symbol	Dimensionless value
Fluid density	${\rho }^{f}$	1.0
Solid density	${\rho }^{s}$	2.0
Gravity acceleration [x, y, z]	${g}_{i}$	[0, 0, 1]
Background permeability	${\kappa }_{0}$	1.0
Fluid viscosity	${\mu }_{f}$	1.0
Bulk compaction viscosity	${\eta }_{C}$	1.0
Background porosity	${\varphi }_{0}$	0.01
Background solid shear viscosity	${\mu }_{0}$	1.0
Carman-Kozeny power-law exponent	${n}^{perm}$	3
Compaction/Decompaction bulk viscosity	$R$	500
Shear viscosity power-law exponent	$n$	3
Effective pressure transition zone	${\lambda }_{p}$	0.01

### Solving strategy

We reached an implicit solution of the coupled set of equations using an iterative pseudo-transient relaxation approach. Acceleration of the residual convergence rates was achieved via a problem-specific damping strategy^46–50^. The numerical algorithm was written in C-CUDA and ran on Nvidia GPUs to efficiently process in parallel 0.25 billion grid points. We relied on Message Passing Interface (MPI) libraries to parallelise the application on distributed memory machines and supercomputers. We calculated the presented high-resolution numerical results in a five-day run on 128 GPUs (Nvidia GTX Titan X Maxwell) in parallel on the *octopus* supercomputer, in-house designed for such high-performance computations, hosted by the Swiss Geocomputing Centre, Institute of Earth Sciences, University of Lausanne, Switzerland.

### Scaling relationships

We used three independent scales:

13 $${\delta }_{c}=\sqrt{{k}_{\varphi }\frac{{\eta }_{C}}{{\mu }_{f}}},$$

14 $${p}_{c}=({\rho }^{s}-{\rho }^{f})g{\delta }_{c},$$

15 $${\tau }_{c}=\frac{{\eta }_{C}}{{p}_{c}},$$

and their dependent combinations such the characteristic velocity  ${v}_{c}={\delta }_{c}/{\tau }_{c}$ to normalise all the variables, resulting in a dimensionless form of the governing equations. The characteristic length scale ${\delta }_{c}$ is also referred to as the compaction length^51^. ${\tau }_{c}$ is the characteristic time and ${p}_{c}$ is the characteristic pressure or stress (buoyancy force). Using the scaling relations (equations (13–15)), the dimensionless model results were scaled to dimensional values representative of reservoir-type rocks: shale, limestone and sandstone. The spacing and the width of the chimneys were mainly controlled by the compaction length ${\delta }_{c}$. The effective size and propagation speed of high-permeability chimneys ranged from centimetre-sized to metre-sized features in low-permeable shale to features in the hundreds of metres in permeable sandstones. The propagation speed varied from 30 centimetres per year in shale representative for caprock to 1 kilometre per year in permeable sandstones (Table 2).

**Table 2:** Scaling of the numerical results to values for reservoir rocks^37,39,52^.

Description	Symbol	Shale	Limestone	Sandstone	Units
Buk viscosity	${\eta }_{\varphi }$	1e13	1e15	1e16	[Pa.s]
Permeability	${\kappa }_{\varphi }$	1e-19	1e-16	1e-15	[m^2^]
Fluid viscosity	${\mu }_{f}$	8e-4	8e-4	8e-4	[Pa.s]
Chimney width		0.1–1	10–100	100–500	[m]
Propagation speed		0.3	300	1000	[m/yr]

## Electronic supplementary material

Supplementary Information guide

Movie 3

Movie 4

Movie 5
