# Supplementary material for: Spontaneous formation of fluid escape pipes from subsurface reservoirs
Source: Sci Rep. 2018 Jul 24;8:11116. doi: 10.1038/s41598-018-29485-5 (PMC6057943; doi:10.1038/s41598-018-29485-5)
Supplement: Supplementary file 1 — Supplementary Information guide [file 41598_2018_29485_MOESM1_ESM.docx]

**Spontaneous formation of fluid escape pipes from subsurface reservoirs**

Ludovic Räss*^1,2^, Nina S.C. Simon^3^ and Yury Y. Podladchikov^1,2^

^1^ Institute of Earth Sciences, University of Lausanne, Géopolis, 1015 Lausanne, Switzerland.

^2^ Swiss Geocomputing Centre, University of Lausanne, Géopolis, 1015 Lausanne, Switzerland.

^3^ SignificaNS, Oslo, Norway.

**Supplementary Information Guide**

**Supplementary Movies:**

**Figure3_movie** Animation of the Fig. 3 displaying a movie of the 100 outputs from the numerical simulation (10’000 physical time steps)

**Figure4_movie** Animation of the Fig. 4 displaying a movie of the 100 outputs from the numerical simulation (10’000 physical time steps)

**Figure5_movie** Animation of the Fig. 5 displaying a movie of the 100 outputs from the numerical simulation (10’000 physical time steps)
